# New trends of additive manufacturing using materials based-on natural fibers and minerals : A systematic review

**DOI:** 10.1016/j.heliyon.2025.e41993

**Published:** 2025-01-16

**Authors:** Joao Ribeiro, Manuel Rodríguez-Martín, Joaquín Barreiro, Ana Fernández-Abia, Roberto García-Martín, Joao Rocha, Susana Martínez-Pellitero

**Affiliations:** a Polytechnic Institute of Bragança, ESTIG-IPB, Bragança, Portugal; b Department of Mechanical Engineering, Universidad de Salamanca, Salamanca, Spain; c Area of Manufacturing Engineering, Universidad de Leon, Leon, Spain; d Centro de Investigação de Montanha (CIMO), Instituto Politécnico de Bragança, Campus de Santa Apolónia, Bragança, Portugal

**Keywords:** Additive manufacturing (AM), Manufacturing processes, Composites, Natural fibers, Mineral additives

## Abstract

Polymeric materials based on natural fibers and minerals are currently being researched and their development is still in its infancy but is expected to increase in the coming years (being nowadays a hot topic). Their main advantage is that they make it possible to use waste and by-products of agricultural, forestry, and mineral origin to generate materials for Additive Manufacturing. Since their use reduces the need for other synthetic polymers derived from petroleum and other non-natural fibers that generate a high environmental impact, this type of material is a sustainable, environmentally friendly, biodegradable solution that can be integrated into the value chain of certain industries and, finally, favors the circular economy. This study presents a bibliometric analysis, meta-analysis, and systematic literature review focusing on plant-based fibers and minerals in biocomposites from a holistic perspective. To learn about the potential of these new materials at an industrial level and to learn about the benefits they can have for society, the strengths, weaknesses, opportunities, and threats have been evaluated. The results strongly suggest that these materials will undergo intensive development in the upcoming years, with a substantial increase in their integration across industries.

## 1 Introduction

Over the past few years, numerous companies have been leveraging Additive Manufacturing (AM) and have started to witness tangible business advantages resulting from their investments [1]. Nowadays, AM technology has matured significantly and has successfully permeated various markets. Its employment spans prototyping and distributed manufacturing, facilitating AM adoption among prospective users. Gradually, AM is reclaiming its position as a valuable asset for enhancing internal productivity, emerging as one of the most prevalent and promising developments in the realms of design and marketing [2]. Furthermore, AM makes circular design strategies possible in the context of a circular economy [3].

AM has gained significant traction due to its inherent simplicity in operating procedures [4]. Currently, AM is integrated within Industry 4.0 and can be combined with other disruptive technology [5]. Using AM reduces material consumption during part production and effectively eliminates material waste. Among the widely recognized AM processes, the Material Extrusion (MEX) technique with composite materials or thermoplastic polymers (coded as MEX-TBR/C P and MEX-TBR/P respectively in Ref. [6]) has emerged for printing intricate designs. This additive technique is also known as FDM (Fused Deposition Modelling) or FFF (Fused Filament Fabrication). However, a common issue with pure thermoplastic-based products is their limited strength and durability, which frequently classifies them for use in prototyping applications across diverse sectors. Additionally, standardizing all the possible available materials can be challenging.

In the MEX-TBR/P technique, a molten material is extruded onto a heated build platform, allowing for extensive customization of various parameters. These include a print line or raster orientation and angle, infill density, printing layer height, print speed, bed temperature, nozzle temperature, and others [7]. To address this limitation and enhance the mechanical properties of thermoplastics, a notable solution has been the incorporation of reinforcing elements, aimed at strengthening the overall composite structure. These reinforcing elements are typically added to the base polymers, resulting in the formation of composite materials. Composites have a structure of polymers, acting as the matrix including constituents commonly referred to as reinforcing components. These can be in the form of continuous fiber or non-continuous fiber. The combination of polymers and reinforcing elements allows for the attainment of enhanced mechanical and thermal properties, surpassing the limitations of pure thermoplastics. Usually, the reinforced fibers most used are carbon fiber. These have very adequate mechanical properties, but they are non-ecofriendly and present difficulties for recycling and biodegradation.

Currently, natural fibers (plant and animal) and particles or powder (minerals) can be combined with different polymer matrixes [8,9]. Since LCA (Life Cycle Assessment) is an important subject to support the development of a Circular Economy [3] the use of natural fibers generates bio-composites, which allows the reduction of CO_2_ emissions and also allows the use of natural resources, favoring the circular economy since it is possible to provide an effective solution to the use of raw materials, since the percentage of the waste is reduced compared to traditional processes and, in some cases, eliminating the waste. In particular, the MEX-TBR/P is a cheap process capable of manufacturing parts without almost waste. In addition, the use of recycled polymer filaments and the use of biodegradable polymeric materials, and biocomposites has intensified in recent years, helping to support the objective of the circular economy.

In the context of the MEX process, this article provides a comprehensive overview of biocomposites utilized to increase the capabilities of printed parts. Specifically, the focus lies on the integration of natural fillers as the reinforcing component. Natural fillers, derived from renewable resources (vegetal and mineral), have garnered attention due to their potential to improve the mechanical performance and sustainability of the resulting composites. Also, some based-on animal resources fiber can be used to generate composites for 3D printing [10].

By shedding light on the potential of biocomposites, this work contributes to expanding the scope of applications for AM technologies, particularly MEX-TBR/P. The findings and insights derived from this research have implications for industries seeking to optimize the mechanical properties and functional capabilities of MEX-produced parts, while simultaneously embracing sustainable and eco-friendly material options.

## 2 Bibliometric and meta-analysis

**Fig. 1:**
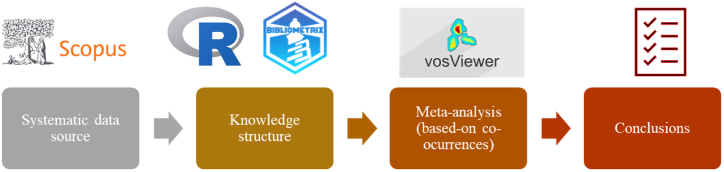
Workflow following the bibliometric and meta-analysis.

In the present work, a systematic bibliographic search methodology is applied, and the data and meta-data obtained are processed using advanced statistical approaches (as illustrated in Fig. 1). For this aim, bibliometric and analytical tools were applied. The overarching objective is to extract meaningful information and elucidate the trajectory of AM of biocomposites within the framework of sustainability.

The datasets of publications were retrieved from the renowned scientific database, Scopus. This dataset encompasses all the necessary information to retrieve data for each document found. The dataset includes all the necessary metadata and details for each publication. Since the goal of the work is related to the 3d printing of biocomposites, the search parameters used are oriented to combinate these words and their synonyms and were included through the following syntax where various keywords with similar practicality were utilized as "or” criteria:

((TITLE-ABS-KEY (additive AND manufacturing) OR TITLE-ABS-KEY (3d AND printing)) AND (TITLE-ABS-KEY (biocomposite)) OR TITLE-ABS-KEY (biocomposites)).

The initial dataset consisted of 437 scientific publications. The dataset was analyzed using R, specifically the oriented-to-bibliometric analysis library Bibliometrix [11]. The summary of the analysis is shown in Table 1. The number of authors reported was 1875. The main document type was the scientific paper (as it should be usual) and the average citation per document was 18.36. All data lead to the conclusion that this is a recent and largely unexplored scientific field.

**Table 1:** Main information about the dataset extracted from Scopus.

Description	Results
Timespan	2004:2024
Sources (Journals, Books, etc)	217
Documents	437
Annual Growth Rate %	5,65
Document Average Age	2,05
Average citations per doc	18,36
References	23415
**Document Contents**	
Keywords Plus (ID)	3192
Author's Keywords (DE)	1071
**Authors**	
Authors	1875
Authors of single-authored docs	8
Single-authored docs	9
Co-Authors per Doc	5,41
International co-authorships %	31,12
**Document Types**	
Article	308
Book	4
Book chapter	19
Conference paper	46
Conference review	10
Editorial	1
Review	49

**Fig. 2:**
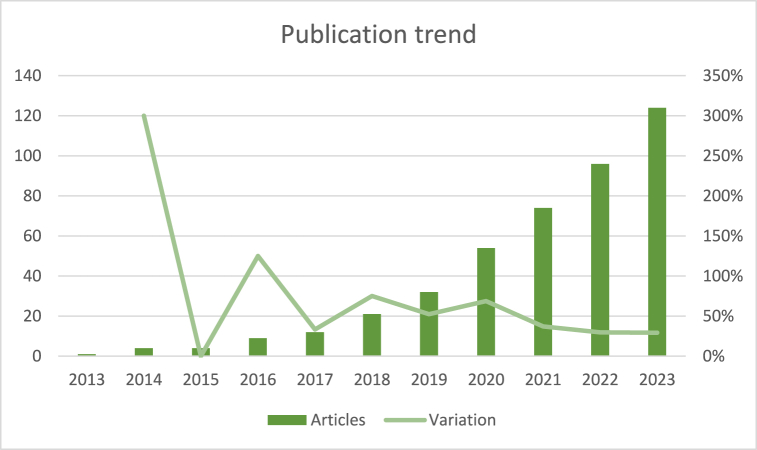
Evaluation of the publication volume in the time (segmented by journal).

After implementing a comprehensive review of the data, an examination of the temporal pattern concerning the quantity of articles (referred to as volume) was conducted using the dataset. The percentage change for each year was computed relative to the preceding year. This analysis reveals a noticeable rise in the volume of articles (Fig. 2). The rate of growth significantly surpasses that observed in the overall number of articles recorded in Scopus (around 37 %) during the same timeframe being compatible with the trends detected for the topic of biocomposites in other works [4]. Nevertheless, despite the evident upward trend, there is a relative deceleration in the pace of increase. This subject was also detected in the articles dealing with AM [12].

**Fig. 3:**
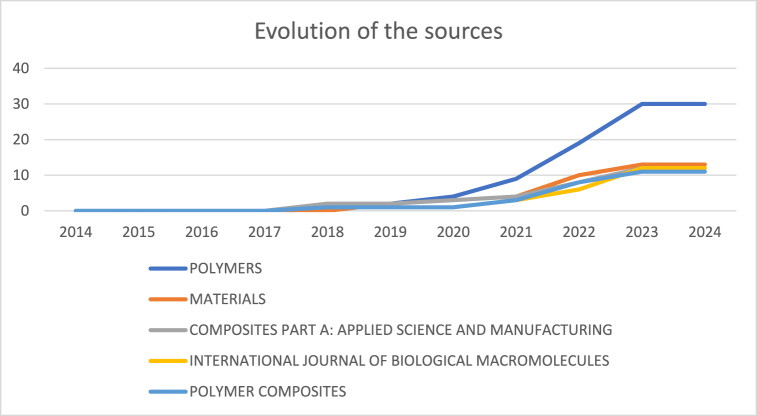
Evaluation of the publication volume in the time (segmented by journal).

The main sources were individually analyzed to evaluate the trend in publication volume (Fig. 3). The main sources that published papers related to the search criteria were Polymers, Materials, Composites Part A, Int. Journal of biological macromolecules and polymer composites. The first of them (highly specialized in polymers) was the source with a higher growth while the second one was a wide-scope journal in material sciences.

Once the publication's trend had been studied, a based-on keyword meta-analysis was conducted using advanced approaches based on the Bibliometrix R library [11] to generate the word map, trend topic map, and thematic map and Vosviewer V1.6.17 to generate the network map [13].

**Fig. 4:**
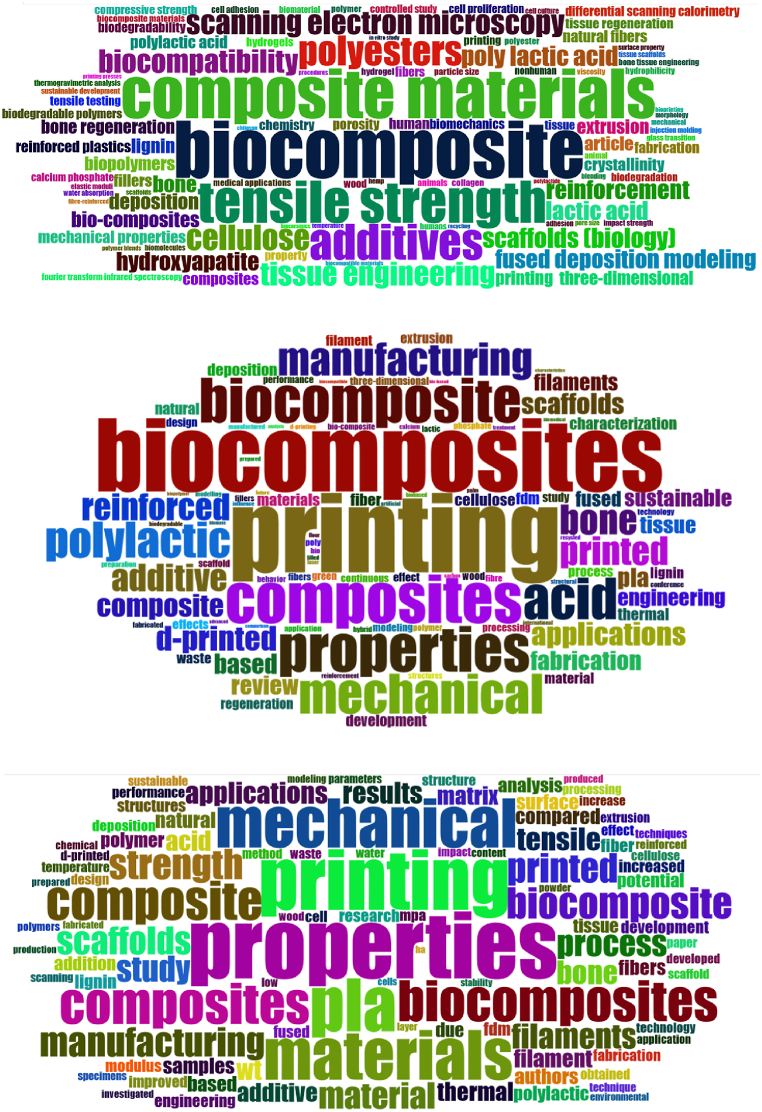
Word map generated from the keyword plus of each document (up), from abstract documents (middle), and from the titles of the documents (down).

The first time, the word map (Fig. 4) was generated for the 100 main words (based on keywords plus, abstracts, and document titles). An exclusion criterion was applied to remove the word AM and its synonymous and redundant words. Fig. 4 shows that the more frequent keywords are related to composite materials, additives, and mechanical properties.

**Fig. 5:**
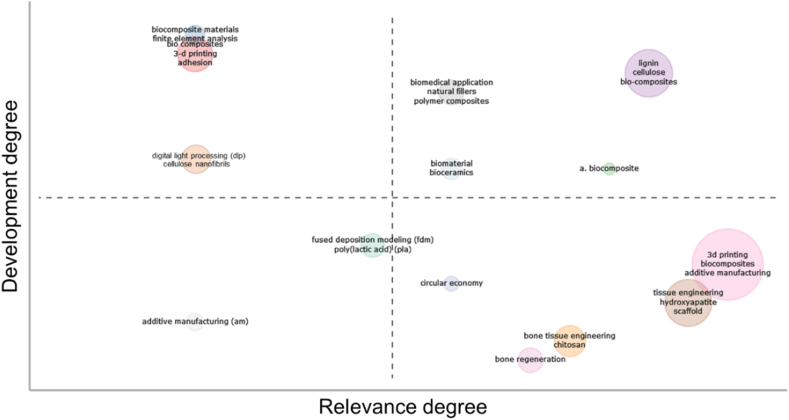
Thematic map applied to keywords.

**Fig. 6:**
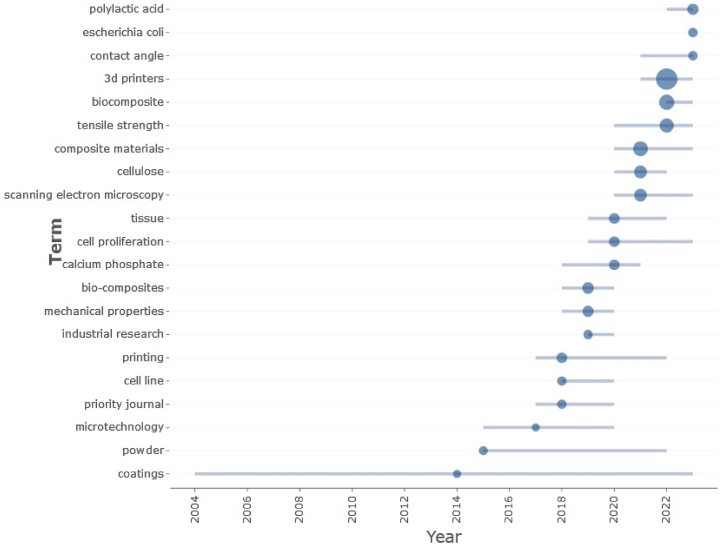
Trend topic map applied to keywords.

Additionally, a thematic map was generated from the keywords (Fig. 5). In this type of visualization tool, each keyword (as topic) is represented on a 2D plot in which the centrality and density are respectively represented. The centrality shows the importance of the keyword within the research field while the density shows the measure of the development of the topic. It used the well-known Walktrap clustering algorithm to create this diagram. The motor and niche themes (first and second quadrants) are directly related to biomaterials and related biomedical and bioengineering use words. Furthermore, research revealed the circular economy to be a high-relevance topic with a low degree of development (Fig. 5). It demonstrates the research potential of this topic, and this is compatible with the conclusions drawn from the trend topic map shown in Fig. 6 where it is shown that the related to biocomposites for AM topics presents are extremely young.

Finally, a meta-analysis was implemented through network mapping. Afterward, clustering techniques were applied to create a co-occurrence network matrix, facilitating the generation of a network map. Before this, the system will refine the keyword set for clustering to prevent word redundancy and analyze relationships between fields. The normalization strategy by fractionalization was chosen for clustering. To simplify the network and prevent irrelevant connections from being searched, the authors established the reproduction of only those keywords that are repeated at least 20 times as a criterion. For both cases, 10 iterations were applied, and small clusters were merged. The goal is to produce a meaningful number of clusters with sufficient link strength and keyword density.

**Fig. 7:**
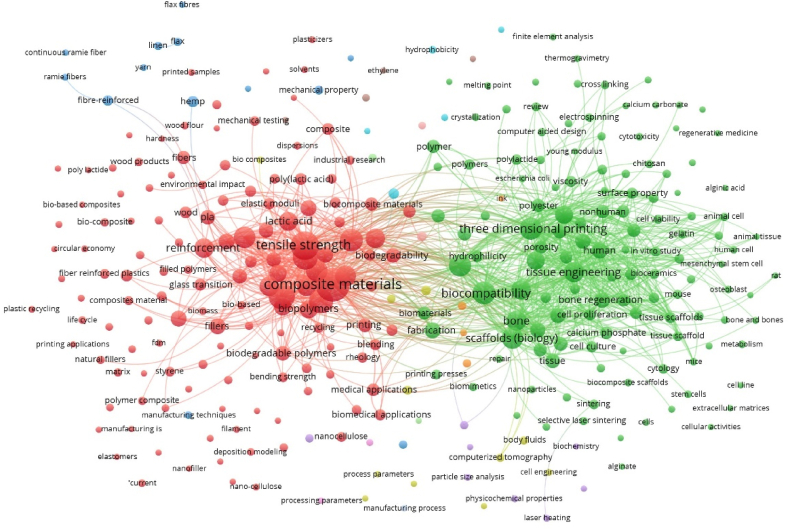
Network map generated from co-occurrence and using the keywords.

In this manner, it is possible to visualize the interrelationships among items (Fig. 7), directly associated with AM and biocomposites. This visualization allows the generation of distinct clusters for assessing the taxonomy within the research field. By employing these strategies, two main clusters will emerge based on keyword relationships. The first (red) has more strength of the link and this is related to the engineering and science of biocomposites, 3D printing, and recycling. The second cluster (green) is also significant, and this is highly related to application for health science and the use of materials in bioengineering. The two clusters showed clear characteristics of their own, being significantly independent of each other.

## 3 Additive manufacturing materials reinforced with natural fibers and minerals

The addition of reinforcements inside the polymer filaments has opened other interesting ways of development. The use of reinforcements improves mechanical properties and, therefore, expands the possible applications of MEX-manufactured parts. Several studies show how the mechanical properties of polymers improve when additives are added as reinforcement. In Refs. [14,15] it is made a detailed review of functional fillers in filaments for AM (ceramics, minerals, metals, natural fibers). From a sustainable point of view, natural fibers and ceramic particles from mineral waste can be two very interesting alternatives to generate biocomposites.

Natural fiber-reinforced degradable polymers could replace the conventional petroleum-based polymers reinforced with synthetic fibers.

The primary objective of incorporating natural fibers in polymer matrix composites is to attain superior thermal insulation, stiffness-to-weight ratio, biodegradability, recyclability, and CO2 neutrality when compared to conventional alternatives such as carbon and glass fibers and, at the same time, to minimize the cost of feedstock materials [16]. There are many AM technologies; however, the most popular are those used as feedstock polymer materials, which can be biodegradable or not. Usually, these polymer materials have relatively low mechanical strength, and to improve this property, it has been tried to reinforce the polymeric base materials with fibers, natural or synthetic. So, the most adaptable AM technologies to add reinforcements are Material Extrusion (MEX), direct-write (DW), stereolithography (SLA), and selective laser sintering (SLS) [17].

### 3.1 Additive manufacturing and composites

As previously referred, the most popular technique used to fabricate polymer composites is the MEX. This process involves melting the feedstock material, which is in the form of a filament. The melted material is then extruded through a nozzle and deposited on a build sheet, following a specific path determined by the 3D CAD model, in order to create the final part [17]. Frequently, used amorphous thermoplastics (PLA, ABS, PC, TPU, PEEK, among others) are applied in MEX because of their wide temperature range and high viscosity, allowing for convenient processing using extrusion nozzles with a diameter of 0.2–0.5 mm [18]. Nevertheless, MEX and other AM technologies are more popular for processing components with a single polymer (in filament or pellets). Specifically, for MEX the polymer most used is the PLA (polylactic acid). PLA is a malleable polymer derived from sustainable agricultural waste through fermentation into a carboxylic acid [19]. The lactic acid undergoes polymerization using a cyclic dilactone called lactide, resulting in a modified product [20]. The outstanding barrier efficiency and good mechanical properties of PLA make it suitable for creating biomaterials for different uses [21]. The limitations of PLA, such as its low resistance to impact, inherent brittleness [22], and susceptibility to water damage, can be effectively enhanced by incorporating fibers and/or fillers. This is a convenient method to enhance the overall properties of the PLA polymer [23].

Polymer matrix composites have been produced using AM techniques, incorporating various reinforcing materials such as particles (at micro and nanoscales) and fibers (both short and continuous). For the MEX technique, both types of reinforcing materials can be incorporated into the filaments of the matrix material (amorphous thermoplastics like PLA, ABS, and PC, among others).

**Fig. 8:**
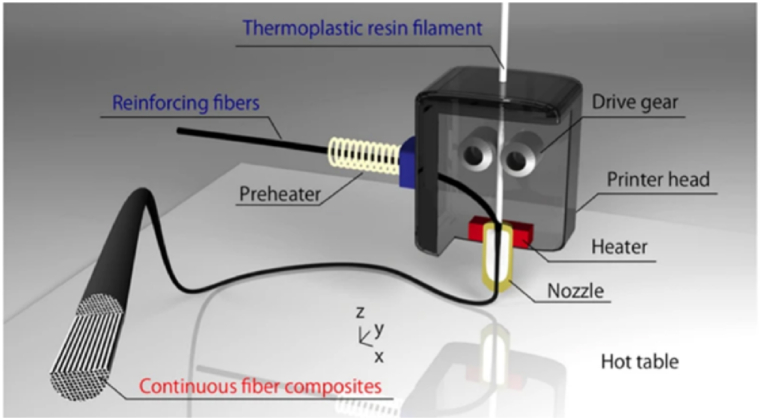
3D printer nozzle employed to produce continuous FRTPs through in-nozzle impregnation, utilizing the MEX technique. Retrieved from Ref. [26].

Fig. 8 presents a schematic representation of how to produce continuous fiber-reinforced thermoplastic polymers (FRTPs) through in-nozzle impregnation, utilizing the MEX technique. Usually, the reinforcements are synthesized, and some examples of particles are W, Fe, graphene, and carbon nanotubes, while examples of fibers are continuous carbon fiber, carbon, and graphene oxide [18]. The most important advantages are simplicity, strong increasing, multi-material deposition, and low cost; however, there are important drawbacks like nozzle clogging and wear, inter-raster porosity, anisotropy, and wavy surface finishes [24]. The matrix and reinforcing fiber combine during the MEX processing of polymer composites to form a composite filament. It is important to emphasize that MEX composite parts possess superior mechanical properties in comparison to compression molding (traditional manufacturing process), enabling their direct utilization as functional components [25]. These advantages led to significant research work on composite polymers reinforced with synthetic materials (Fig. 8) using different AM technologies [17]. Nevertheless, the use of these AM technologies for the processing of natural fibers-reinforced composites (NFRCs) is exceedingly uncommon.

### 3.2 Natural fibers for additive manufacturing

The NFRCs have gained significant recognition in recent years due to their exceptional performance and environmentally friendly nature [27]. Oksman and colleagues, for instance, developed an environmentally friendly composite material in the early 21st century. They combined flax fiber and polypropylene with polylactide (PLA) as a reinforcing agent and triglyceride as a plasticizer. PLA composite materials exhibit a mechanical strength that surpasses polypropylene (PP) composites by approximately 50 %, resulting in extensive application across various fields [28]. Several researchers have developed new FRTPs using MA in the last few years, in addition to the previously mentioned example. Table 2 showcases a handful of research examples.

**Table 2:** Specifications of filaments in prior research.

Fillers	Matrix	% of Fillers	Diam.	Pre-treatment	Modifiers/Agent	Ref.
Continuous flax and jute	PLA	–	1.8	–	–	[29]
Hemp	Pre_PP	10–30	2.5	MA/2	A + Na_2_SO_3_	[30]
Wood	PLA	0, 10, 20, 30, 40, 50	1.75	–	–	[31]
Sugarcane bagasse	PLA	0, 3, 6, 9, 12, 15	1.75	–	Sodium hydroxide, sodium chlorite, and glacial acetic acid	[32]
Rice husk	PLA/	0, 10, 20, 30, 40	1.75	Tetraethyl orthosilicate	–	[33]
Kenaf powder	PLA	2.5	1.75	–	A + S	[34]

The classification of natural fibers is commonly based on their origin, which includes animal fibers, mineral particles, and plant or vegetable fibers [35]. Particularly, the plant-derived fibers can be classified according to their source and are usually categorized into bast (hemp, flax, jute), leaf (sisal, abaca, banana), fruit/seed (coir, coconut, cotton), grass/reed (bamboo, switchgrass, miscanthus), and agriculture residue (wheat straw, soy hull, corn stover) [36]. These kinds of fibers, in general, are lightweight, having a density of 1.1–1.6 g/cm^3^ [17]. The main components of plant fiber are cellulose (60–80 %), hemicellulose, and lignin (5–20 %). The remaining constituents consist of waxes, pectin, moisture (up to 20 %), and water-soluble organic components [37]. Despite the aforementioned advantages, the mechanical properties of vegetal fibers are typically inferior to those of synthetic fibers (considering the current state of the art). However, through careful surface treatment, it is possible to achieve comparable or even superior mechanical properties in plant fibers compared to synthetic fibers [23]. Typically, some mechanical properties like tensile strength have a range of 20–1600 MPa, Young's modulus of 4–128 GPa, and elongation at the break between 1 and 30 % [38]. The value of these properties depends on plant fiber composition, crystallinity, microfibril angle, and internal structure [39] and, for each plant, it also depends on sowing conditions [40].

The AM of composites demonstrates evident and robust potential in the production of complex parts. However, to disseminate these technologies it is necessary to overcome some issues, namely, challenges in the production of composite feedstock filament for MEX including void formation, nozzle clogging, clumping and distribution of fibers, the impact of fibers on the curing process, and resolution, alignment of fibers, and the adhesion between fibers and matrix [17].

As in any NFRC, the primary difficulty in this kind of composite is the adhesion between the fibers and matrix due to the nature of the materials. Typically, the polymer matrix has a hydrophobic nature while natural fibers have a hydrophilic nature and consequently their wettability with polymer matrices is very poor. These characteristics also impact the distribution of fibers within the matrix, causing it to become non-uniform due to the incompatibility between the fibers and the matrix. Some researchers observed other drawbacks caused by the low interface adhesion composite-matrix, such as a large number of interfacial porosities resulting in an important reduction in tensile strength [41], enhancing the capacity of composites to absorb moisture, resulting in residual stresses, swelling, intensified biological degradation, and weakened strength [42]. Hence, the critical initial stage in the production of NFRCs involves the pre-processing of natural fibers before their integration into polymer matrices. This pre-processing entails two key procedures: (a) surface preparation of the natural fibers, and (b) modification of the polymer matrix through the addition of compatibilizing agents. There are different techniques to modify the surface of natural fibers but the most common are the physical, chemical, and biological and these treatments aid in eliminating impurities from the fiber surface and minimize the hydrophilicity property while growing fiber/matrix uniformity [43,44]. Matrix modification is a widely used method to enhance the interactions between fibers and matrix. This involves chemically modifying the matrix using maleic anhydride (MA) and adding maleated polymer to the matrix [17].

An essential factor to consider in the AM of NFRCs is that none of the existing techniques apply pressures and shear rates that are sufficiently high for the material. Consequently, the composites lack robust interlayer bonding in AM-processed parts, leading to subpar mechanical properties, particularly in the transverse direction (where the loading axis is parallel to the build direction). The structural integrity of these components is primarily derived from the bonding that occurs through thermal diffusion between the interlayers and the roads. Consequently, the components produced using AM technologies frequently lack high mechanical strength. A possible solution for improving the low value of mechanical strength is to use feedstock materials with a high concentration of reinforcements and minimal porosity; however, an excessively high concentration of fibers can lead to an increase in the composite's viscosity and cause-related issues [17].

There are multiple challenges associated with the development of composite feedstock filament for MEX [4]. The inherent process steps of blending, compounding, and extrusion used in the preparation of feedstock composite filaments can cause damage to natural fibers because of exposure to high temperatures and pressures. In addition, the fibers undergo elevated temperatures during MEX processing, which can further degrade their properties and characteristics. As previously mentioned, as the fiber concentration increases, the viscosity of the polymer also increases. Consequently, in order to facilitate the smooth processing of NFRC filaments, it is necessary to raise the extrusion temperature. However, this increase in temperature may have negative effects on the stability of natural fibers.

**Fig. 9:**
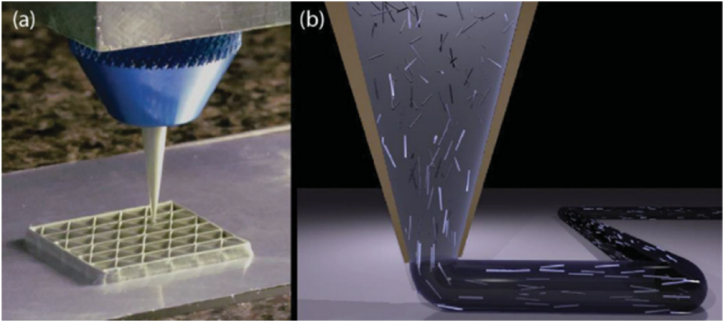
Triangular honeycomb obtained by AM (a) and the schematic representation of 3D printer nozzle utilized for creating continuous FRTPs through in-nozzle impregnation, employing the MEX technique. Retrieved from Ref. [46].

An important challenge that needs to be addressed is the severe obstruction of nozzles (Fig. 9) that occurs during the MEX process of fiber-reinforced polymer composites containing more than 40 wt percent of synthetic fibers. Moreover, it has been noted that feedstock filaments exhibit increased fragility when subjected to high fiber loading [45]. Hence, achieving enhanced mechanical properties in fiber-reinforced composites through increased fiber loading would be exceedingly challenging without a comprehensive understanding of the impact of fiber concentration on rheological properties, as well as the interactions between fibers and the matrix during feedstock preparation and AM processing.

**Fig. 10:**
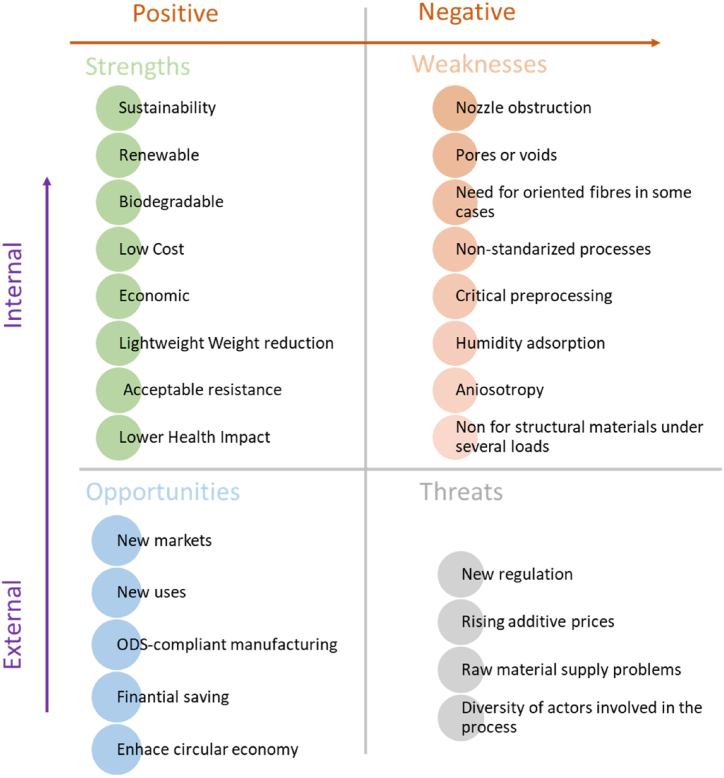
SWOT analyses of the application of vegetal fibers to generate 3D-printable biocomposites.

The presence of pores or voids and the alignment of fibers in natural fiber-reinforced composites significantly influence their mechanical properties. Controlling the fiber orientation within the matrix is crucial during AM processing due to its significant impact on composite properties. To effectively eliminate the negative impact of porosity, it is necessary to align the fibers in the direction of the applied load. Certain AM processes, such as MEX or DW, can customize the alignment of fibers while they are being processed [46], see Fig. 9. A strength-weaknesses-opportunities-threats (SWOT) analysis has been implemented to evaluate the scope of the topic (Fig. 10).

**Fig. 11:**
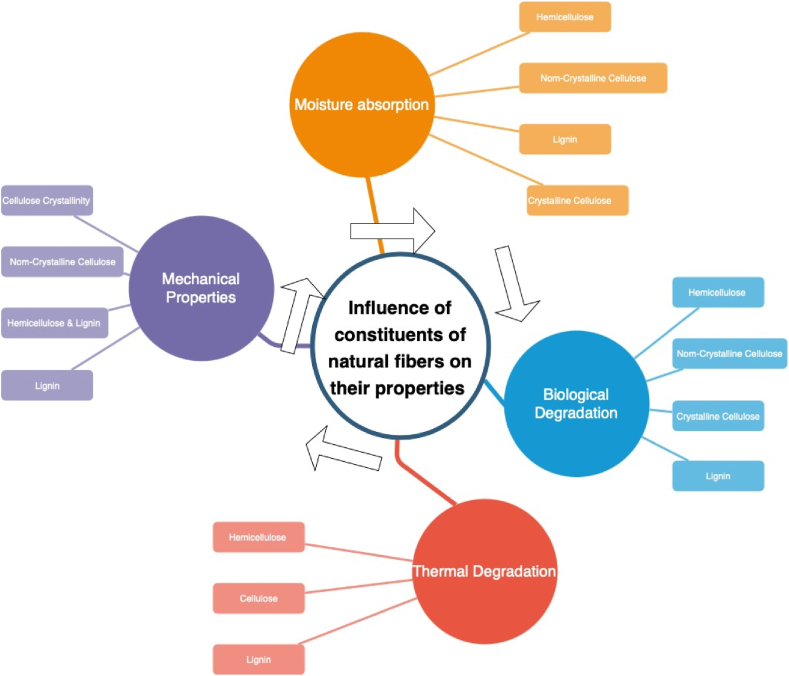
Impact of components of natural fibers on their characteristics (Adapted from Ref. [47]).

Based on the preceding analysis, it is evident that the effective employment of NFRCs through both AM and traditional processing methods is constrained by the inherent properties of natural fibers, which are contingent upon their chemical composition. The relationship between the constituents of natural fibers and their various properties, as depicted in Fig. 11, reveals conflicting compositional prerequisites for attaining desired characteristics such as mechanical strength, thermal conductivity, biological compatibility, and moisture absorption.

### 3.3 Mineral materials for additive manufacturing

The use of ceramic particles as reinforcement in 3D printing polymeric filaments is promising for manufacturing the parts in different applications, such as tool manufacturing or the manufacture of sacrificial patterns for investment casting process (ICP), or in applications of controlled porosity polymer-ceramic composite scaffolds [48] in fabric applications [49]. A complete and very interesting review can be found in Gomes et al. work [50] with recycled thermoplastic matrices. The review of papers reveals that several studies have already used certain minerals as filament fillers due to their low cost, ease of processing, and significant impact on mechanical and thermo-mechanical properties.

Graphene is a novel and disruptive material composed of a single layer of carbon atoms arranged in a two-dimensional hexagonal structure. It is incredibly strong, lightweight, flexible, and a good conductor of electricity. Scientific studies have shown that the addition of graphene to ABS and PLA filaments increases the mechanical strength of 3D printed material, providing higher durability and rigidity to the manufactured parts [51,52]. In addition, graphene's electrical conductivity opens up new possibilities for specific applications, such as the manufacture of electronic components integrated directly into printed parts. This has significant implications for the electronics industry and the development of more efficient and functional devices [53,54]. In addition, the thermal conductivity of graphene contributes to better heat dissipation by prolonging the lifespan of electronic devices [55]. While it is essential to consider aspects such as graphene production and recycling in terms of sustainability, its ability to improve material properties and extend the life of 3D printed products can positively contribute to reducing environmental impact in certain applications.

The addition of carbon fibers to polymeric matrices is another alternative to improve the strength and rigidity of 3D printed parts, in short or continuous fiber format. In the case of short fiber reinforced thermoplastic materials, most of the studies focus on developing filaments that incorporate micro and nano-sized fibers. Several papers show the potential of using recycled fibers as a sustainable and economic alternative to virgin fibers in AM applications [[56], [57], [58]]. Although carbon and graphene have been the most researched fillers, other authors focused on other ceramic materials and minerals. For example, Tayfun et al. incorporate pumice in ABS filament [59]. Alghdi et al. [60] added perlite mineral in the ABS matrix and Spoerk et al. [61] in the polypropylene (PP) matrix, concluding that mechanical properties improve and, in the case of PP, also the warping effect. In Ref. [62], the authors used bentonite, improving in all cases several properties (mechanical strength, elongation, and hardness) at certain concentrations of the mineral, although in some cases toughness worsened. Other authors use montmorillonite to seek a balance between mechanical strength and ductility in PLA or ABS matrix [[63], [64], [65], [66]]. On the other hand [67], add montmorillonite nanoparticles but, in this case, looking to improve the surface of printed parts (hardness, roughness, and dimensional accuracy).

In [68], the authors added talc powder to a PLA matrix as a nucleation agent to improve crystallization during cooling. Seeking complementary results, in Ref. [69] the authors compare the addition of montmorillonite, biocarbon, and talc as fillers in a PLA matrix. This research concluded that montmorillonite and talc provide better results than biocarbon in terms of mechanical properties, but at the thermomechanical level, no improvements were observed.

In [70,71] it was investigated the influence of silicon dioxide as filler in ABS and PP matrix, respectively, with contrary results in the improvement of mechanical properties at high concentrations (while the elastic modulus improved, mechanical strength, ductility, and toughness worsened) [72]. used hydrated magnesium silicate as a filler in the ABS matrix. In Ref. [73], it was used iron powder together with ABS and TPU as matrix, and [74] used ash from combustion processes, with similar results to previous work. In Ref. [14], the authors incorporate glass powder as an additive in polypropylene-based additive manufacturing filaments, also demonstrating the beneficial effect on mechanical properties as a function of powder concentration. In Ref. [75] similar study was developed but using short fiberglass to analyze the fatigue behavior of the material. Similarly, in Ref. [76] the authors added pearl powder to PLA, to improve the overall performance in terms of mechanical and biological properties in bone implants.

In [77], the authors used zeolites (microporous aluminosilicate minerals) in a PLA matrix, analyzing the effect on the morphology and thermal properties of the filament. In Ref. [78], the authors added silane-treated wollastonite to an ABS matrix, demonstrating an improvement in both mechanical and thermal properties, and extending the degradation of the material at high temperatures.

In [79], it was used a mixture of aluminum powder and alumina in a Nylon6 matrix, looking for a high-strength filament; aluminum was chosen for its lubricating effect and alumina for its abrasive effect. Similarly, in Ref. [80], the authors used alumina and silicon carbide as fillers in Nylon6, trying to achieve properties like those of ABS [81].

In another field, in Ref. [82] or [83] mineral phases such as hydroxyapatite to PLA and PCL filaments were applied, in this case looking for bone compatibility in implants. With the same objective [84], applied the ceramic material biphasic calcium phosphate in PLA matrix.

On the other hand, the use of slate as filler in polymeric composite materials is scarce. Slate is a metamorphic rock with a composition based on quartz, phyllosilicates, illite, and other minor components [85]. The slate residues in quarries contain significant amounts of all materials in the aforementioned research: silicon dioxide, iron oxide, aluminum oxide, calcium oxide, titanium oxide, magnesium oxide, etc. [86]. Therefore, the slate is an interesting material for using as filler component in filaments for AM.

Slate is a widely used material in construction, especially for roofs, walls, and floors. The slate quarries and processing plants generate a lot of waste [87] representing up to 95 % of the extracted rock, whose size varies from rock blocks (>10 cm) to sludge. Most of the waste generated is sent to landfills, causing an environmental problem. Spain is the world's leading producer of slate [88]. In Spain, Castilla y León and Galicia regions are the largest producers, with different types of slate [89,90].

Slate residues have been used in several other applications as fillers due to their availability of low cost: as a cement component [91,92], as reinforcing fiber for high-density polyethylene [86], in the production of bricks [93], in automotive brakes [94], etc. However, slate has not been almost studied as reinforcement in polymeric filaments for AM. In the review carried out, only a research group was found, that uses Multanpura slate (India) in filaments for MEX additive manufacturing: in PLA filament [95] and ABS filament [96].

**Fig. 12:**
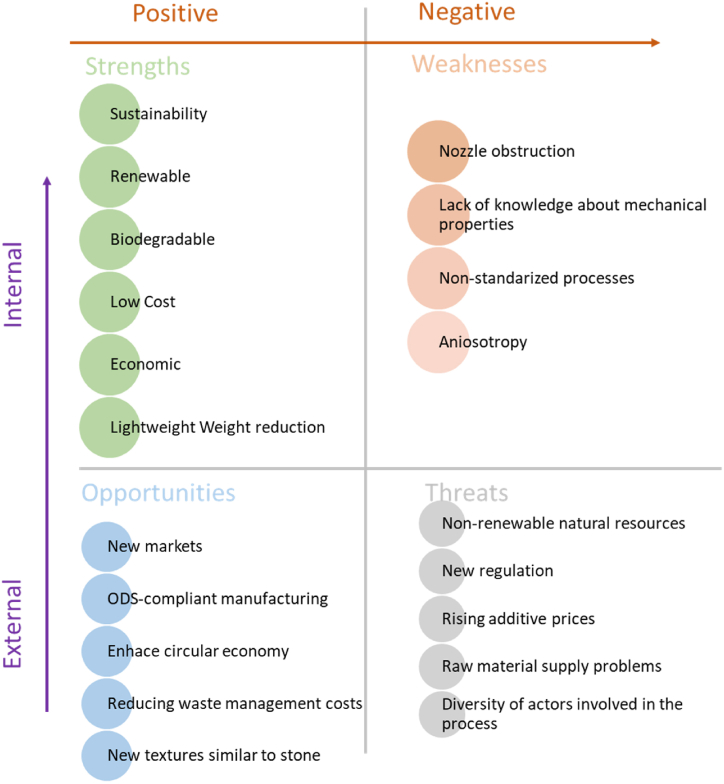
SWOT analysis applied to the use of mineral reinforcement for the generation of biocomposites for AF.

Moreover, it should be noted that, in all the previous studies, the research focuses on the analysis of mechanical or thermomechanical properties of filaments with different filling concentrations (maximum of 15 %). Despite being of great interest, two main aspects are not analyzed: a) the parameters and operating strategies during 3D printing to ensure reliable use of this process with these materials; b) the dimensional, geometric, and surface quality of the printed parts. These are necessary aspects, complementary to the analysis of mechanical properties. In conclusion, the satisfaction of a work niche necessitates further knowledge transfer to society and industry. Unlike vegetable fibers, this case presents a challenge in defining its scope and potential development, as it is still in its infancy. However, we can establish a SWOT analysis using the evidence from this research and the similarities with vegetal fiber-based biocomposites. Continuous development although the SWOT analysis (see Fig. 12) can be established based on the evidence found in this research and based on the similarity with the biocomposites based on vegetal fiber.

## 4 Discussion

By encouraging a circular economy, sustainability, and lowering dependency on non-renewable resources, the current state-of-art demonstrates that the incorporation of natural fibers and mineral fillers into additive manufacturing (AM) represents a substantial advancement towards sustainable production techniques. In this study, the benefits of employing plant-based fibers (especially when creating biocomposites) have been documented. Their biodegradability, renewability, and low density make them appealing substitutes for the synthetic fibers frequently found in polymer biocomposites. Comparably, fillers based on minerals offer special qualities that improve the mechanical and thermal performance of materials that are 3D printed, despite their less research with the disadvantage that they are non-renewable resources but at the same time they are also wastes that could not be used for other purposes.

On the one hand, considering that there exist different parameters that influence the quality of the final material (moisture absorption, mechanical properties of the fibers, etc.) the fact that adequate surface treatment and fiber alignment can ameliorate the decreased mechanical strength of vegetal fibers relative to synthetic fibers is one of the important findings. It has been demonstrated that the mechanical capabilities of biocomposites can be enhanced to a level that could become comparable to synthetic alternatives (consolidated) by specific treatments and modifications. Moreover, the utilization of agricultural leftovers for reinforcement not only tackles environmental issues but also creates novel prospects for enterprises in search of environmentally sustainable material solutions.

The scalability of these biocomposites is another crucial factor to consider. Although they have a lot of potential for usage in consumer goods, biotechnology, and construction, their industrial acceptance is now limited by the absence of standardized material qualities and processing methods. It will be essential to overcome these technological obstacles in order to translate research findings into practical applications, especially in maintaining consistency in fiber-matrix interaction and optimizing printing parameters.

On the other hand, mineral fillers are common in AM materials when special characteristics with respect to the usual materials are desired. Graphene or carbon fiber are fillers that have been widely investigated. However, they are polluting resources, although in the case of carbon, possibilities of using recycled fiber are being investigated. Other mineral fillers have also been investigated but not with the same intensity as the previous ones. However, mineral fillers from mining waste, such as slate, have hardly been investigated and their use represents an improvement in sustainability because it allows the industrial use of a waste product, while at the same time saving the costs derived from its elimination. Since this research is in its infancy, it is necessary to do more thorough testing to standardize material compositions and processing conditions for composites reinforced with mineral waste.

Based on the implemented research, it can be concluded that biocomposites made from vegetal fibers and those composites using mineral waste fillers represent emerging fields in advanced material science and AM. However, their levels of development differ significantly. In the case of plant fiber-based biocomposites, while further research is needed to explore the impact of different parameters (e.g. filler type, surface treatments to enhance adhesion and cultivation methods), several studies already provide insights into their mechanical properties and potential applications. In contrast, composites using mineral waste fillers remain largely under-researched, with only a limited number of studies addressing their performance. This gap highlights a significant challenge and opportunity for future research and development in this area.

Finally, based on the above, Table 3 shows a comparison between composite materials for 3D printing based on natural fibers and synthetic fibers.

**Table 3:** Non-exhaustive comparison between 3D-printable composites based on natural and mineral fibers and synthetic composites.

Criteria	Natural/mineral resources	Synthetic Fibers
Mechanical properties	Lower tensile strength compared to synthetic fibers (in the current state of art) but can vary significantly based on fiber type. Moderate stiffness: varies between fiber types (e.g., jute is stiffer than cotton). In the case of mineral fibres, further research is needed.	High tensile strength, especially in fibers like glass, carbon, and aramid. High stiffness, especially in carbon and aramid fibers.
Weight	Lightweight; lower density compared to synthetic fibers but depends on the specific material.	Generally, slightly heavier, though fibers like carbon are lightweight.
Thermal Resistance	Generally lower thermal resistance: can degrade at higher temperatures but depends on the fiber type. In the case of mineral fibers, further research is needed.	High thermal resistance, especially for aramid and glass fibers.
Impact Resistance	Moderate impact resistance; depends on fiber type and processing. In the case of mineral fibers, further research is needed.	High impact resistance, especially with glass and aramid fibers.
Aging and Degradation	Susceptible to UV and environmental degradation over time. In the case of mineral fibres, further research is needed.	Less susceptible to environmental degradation; UV resistant in specific types (e.g., carbon).
Moisture Absorption	High moisture absorption that reduces mechanical properties.	Low moisture absorption; more dimensionally stable.
Processing	Easier to process at lower temperatures, with less wear on machinery.	May require higher processing temperatures; can wear down equipment and requires equipment to work safely.
Compatibility with Matrix	Usually requires surface treatments for optimal bonding with polymer matrices.	Typically compatible, especially with thermoplastics, though surface treatments can enhance bonding.
Biodegradability	Biodegradable; aligns with sustainability objectives and circular economy.	Not biodegradable; can persist in the environment for long periods. This is harmful to the environment.
Cost	Generally lower; abundant and renewable sources.	Higher production costs that can vary significantly by type.
Sustainability	High sustainability potential; derived from renewable resources	Lower sustainability; derived from non-renewable sources.
Recyclability	Often more challenging to recycle in composites but easier in pure form.	Can be recycled, though complex in composites due to resin-fiber integration but the recycling process can be complex and expensive.
Availability	Widely available but region-specific. Allows to take the advantage of local resources and waste.	Available, with global supply chains for consistent quality but depends on non-renewable sources.
Environmental Impact	Lower overall impact; reduced carbon footprint and energy requirements. Even is good to ecosystem and rural areas development.	Higher carbon footprint; energy-intensive production processes.
Applications	In the current stat of art, they are ideal for low-load applications with sustainability goals (e.g., automotive interiors, furniture).	Ideal for high-performance applications requiring durability and strength (e.g., aerospace, automotive structures) but it is a polluting and unsustainability.

## 5 Conclusions

Based on the meta-analysis, two main trends were detected within the biocomposites to 3d printing topic: one related to the engineering and science of the materials to improve the characteristics and to create new materials based on new removable/recyclable resources and, on the other hand, another topic related to the application of the materials to bioengineering.

Natural reinforcements that can be used to create 3d printing materials are classified into mineral animal, and vegetable fibers, with the latter being the most common. Vegetable fibers come from various parts of plants and can be categorized as bast, leaf, fruit/seed, grass/reed, and agricultural residues. They have a low density, and their use is sustainable and biodegradable.

Although their mechanical properties are typically lower than synthetic fiber (usually polluting and non-renewable), with proper surface treatment, several experimental works have shown that they can match or even surpass them. However, these properties highly vary depending on the fiber composition, crystallinity, microfibril angle, and planting conditions. For this reason, NFRCs have gained recognition in AM uses for their exceptional performance and eco-friendliness.

Mineral resources are also used as reinforcement in composites for additive manufacturing. Studies have shown contrasting results with the addition of fillers like silicon dioxide and hydrated magnesium silicate, with some observing improvements in mechanical properties while others noting deterioration. Nevertheless, these efforts contribute to expanding the knowledge base surrounding additive manufacturing materials. One underexplored area is the use of slate as a filler in polymeric filaments for AM. Slate, abundant in waste from quarries and processing plants, presents an opportunity for low-cost reinforcement. However, research in this area remains limited, with only a few studies investigating its potential.

Regarding the challenges in AM with Natural Fiber-Based Materials, despite progress in studying mechanical and thermomechanical properties, there is a need to address parameters and operating strategies during 3d printing to ensure reliable use of these based on natural fibers materials. Additionally, attention should be given to dimensional, geometric, and surface quality considerations of printed parts.

From a critical standpoint, it can be seen that materials based on vegetable fibers are biocomposites with all their advantages (which have been listed) and that they have an important relationship, not only with AM but also with health sciences. Materials based on mineral fibers do not have a biological origin, but they can be used to take advantage of mining and mineral resources and improve sustainability and biodegradation in AM, as well as enhance the circular economy and the value chain of the mining and construction industry. Even so, these materials are less researched than biocomposites based on plant fibers in terms of process parameters and mechanical properties and their potential is very intense, representing an opportunity today.

Concerning the potential for industrial systematization of these materials, the results reported in this work are still in the research phase, and there is no direct and general application at the industrial level (because it is a novelty that has only been under investigation for 5–8 years, as demonstrated in the bibliometric analysis). Nevertheless, prospectively, it is possible to predict the advantages and disadvantages that the cited research might have when adopted by companies, which represents a contribution from this work. Therefore, the SWOT analysis reveals a variety of opportunities and challenges for businesses using natural resources as reinforcement materials for additive manufacturing. In general, promoting circularity and sustainability, aligning with the SDGs, and creating new markets for plant fibers through the utilization of by-products always presents an advantage. Thus, each company or industry that intends to take advantage of these resources will have to assess their integration into the manufacturing processes from different perspectives: on the one hand, it will be necessary to know whether the product to be manufactured will have the appropriate properties, for which a quality analysis and testing of the materials may be necessary, as these are materials that are not currently standardized. On the other hand, the suppliers of raw materials are very important, since both vegetable and mineral fibers are endogenous resources whose sources must be analyzed in order to integrate them into the value chain.

For mineral reinforcements, the possibility of achieving textures like natural elements in the final pieces may be of interest to companies looking to create a differentiated product. Finally, the systematic production of these types of materials is not without challenges, particularly concerning new regulations, costs of necessary additives for proper adherence to the reinforcement material, issues with raw material supply chains, and a diversity of factors in the process. Therefore, it is necessary to develop well-defined biotechnological routes to ensure logistical success in the production of these materials.

Concerning environmental benefits and the enhancement of the circular economy, it is clear that vital fiber-based materials are renewable and biodegradable and represent a major breakthrough that has significant benefits in terms of sustainability. Additionally, in the specific case of mineral reinforcements, it is not a renewable resource, although it is indeed a waste with potential for reuse.

Finally, the primary goal of these materials is to advance the circular economy, thereby enhancing sustainability and benefiting the environment. By promoting the use of resources commonly found in rural areas suffering from depopulation and economic challenges, these materials serve an important social purpose.

In conclusion, even though the study and development of new circular materials for 3D printing have made significant strides, more research is required to close the gap between academic findings and useful industrial applications. Continued exploration and innovation in this field will be essential to unlocking the full potential of these materials and their contributions to sustainability, economic development, and social well-being.

## CRediT authorship contribution statement

**Joao Ribeiro:** Writing – review & editing, Writing – original draft, Visualization, Validation, Supervision, Software, Resources, Methodology, Investigation, Funding acquisition, Formal analysis, Data curation, Conceptualization. **Manuel Rodríguez-Martín:** Writing – review & editing, Visualization, Validation, Supervision, Software, Resources, Project administration, Methodology, Investigation, Funding acquisition, Formal analysis, Data curation, Conceptualization. **Joaquín Barreiro:** Writing – original draft, Validation, Supervision, Methodology, Investigation, Formal analysis, Conceptualization. **Ana-Isabel Fernández:** Writing – review & editing, Writing – original draft, Supervision, Investigation, Formal analysis, Conceptualization. **Roberto García-Martín:** Validation, Resources, Project administration, Investigation. **Joao Rocha:** Validation, Supervision, Methodology, Investigation, Funding acquisition, Conceptualization. **Susana Martínez-Pellitero:** Writing – review & editing, Writing – original draft, Visualization, Validation, Supervision, Methodology, Investigation, Formal analysis, Data curation, Conceptualization.

## Data availability statement

No new data was generated for the research described in the article.

## Declaration of competing interest

The authors declare the following financial interests/personal relationships which may be considered as potential competing interests:Manuel reports was provided by European Commission (Interreg-POCTEP). If there are other authors, they declare that they have no known competing financial interests or personal relationships that could have appeared to influence the work reported in this paper.
